# Adolescent Idiopathic Scoliosis – case report of a patient with clinical deterioration after surgery

**DOI:** 10.1186/1754-9493-1-7

**Published:** 2007-12-19

**Authors:** Hans-Rudolf Weiss

**Affiliations:** 1 Asklepios Katharina Schroth, Spinal Deformities Rehabilitation Centre, Korczakstrasse 2, D-55566 Bad Sobernheim, Germany

## Abstract

**Background:**

Although there is no evidence that the long-term effects of scoliosis surgery are superior to the long-term effects of Adolescent Idiopathic Scoliosis (AIS) itself, patients can fear the consequences of not under going this surgery due to incorrect or insufficient information. The main indication for surgical treatment in patients with AIS, is cosmetic. However spinal surgery may, along with other negative side effects, actually cause postoperative clinical deterioration. This complication of surgery has not yet been described in international literature.

**Case presentation:**

A 15-year old female patient originally presenting with a well-compensated double curve pattern scoliosis. The patient was advised to undergo surgery due to the long-term negative impact of signs and symptoms of scoliosis upon her health. The patient agreed to surgery, which was performed in one of Germanys leading centres for spinal surgery. The thoracolumbar curve was corrected and fused, while the thoracic curve, clearly showing wedged vertebrae, defined as structural scoliosis, remained untreated.

This operation left the patient with an unbalanced appearance, with radiological and clinical imbalance to the right. The clinical appearance of the patient though clearly deteriorated post-surgery. Furthermore, the wedged disc space below the fusion area indicates future problems with possible destabilisation accompanied probably by low back pain.

**Conclusion:**

Scoliosis surgery for patients with AIS is mainly indicated for cosmetic or psychological reasons. Therefore the treatment leading to the best possible clinical appearance and balance has to be chosen. Patients should be informed that surgery will not necessarily improve their health status. Clinical deterioration after surgery may occur, and such information is crucial for an adequate informed consent.

## Background

Adolescent Idiopathic Scoliosis (AIS), the most common form of  scoliosis, is a structural three-dimensional deformity of the spine and  trunk, occurring in otherwise healthy children during puberty. Curvatures <  10° are viewed as a variation of normal [1], as those curves have little potential for progression [1]. Historically, in southern and central Europe  the treatment for AIS consists of: physiotherapy (PT) on an out-patient  basis; Scoliosis In-patient Rehabilitation (SIR); corrective bracing and  surgery with or without spinal fusion.  

Scoliosis management is usually regarded as effective when curvature progression has been stopped below a certain limit, although other parameters than progression may play an important role for the individual patient [2,3]. Surgery should only be considered an option for the patient, when all conservative measures have failed.

**Figure 1:**
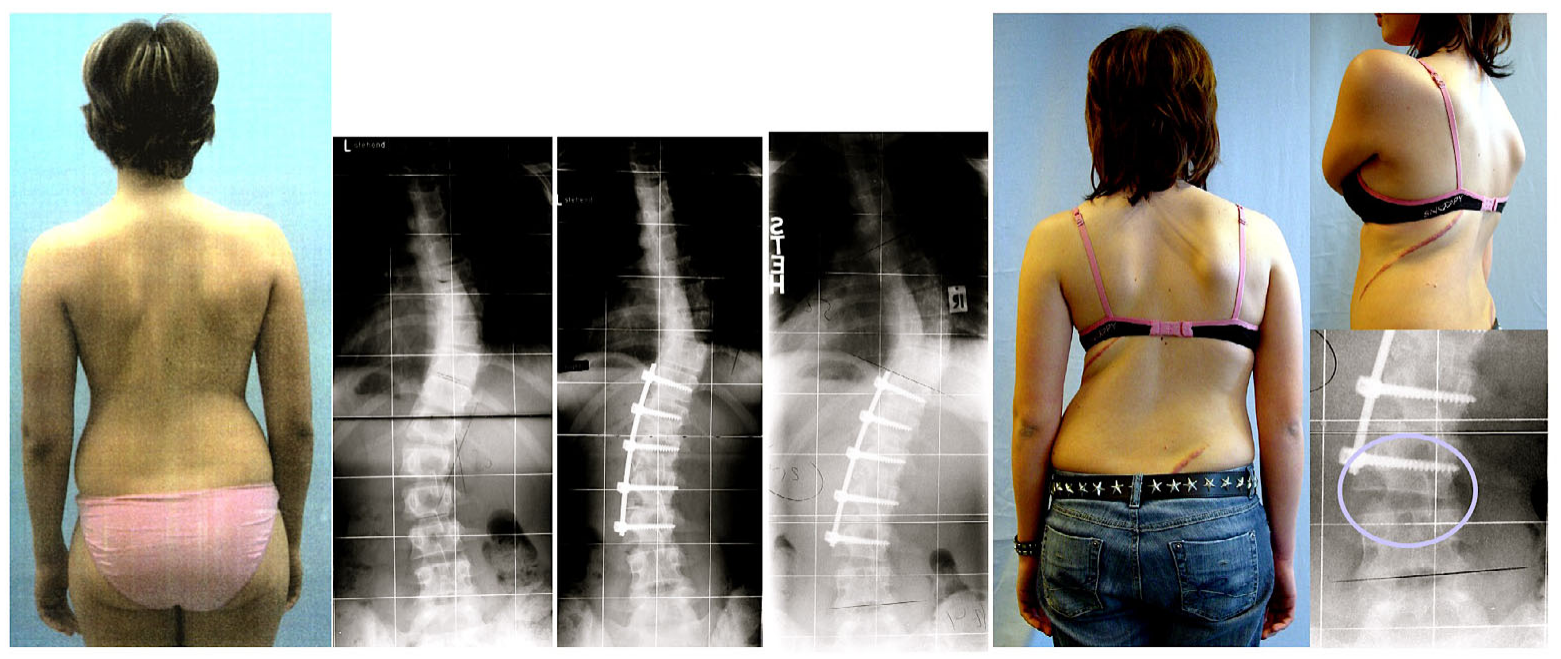
**Clinical deterioration after surgery for Adolescent Idiopathic Scoliosis (AIS)**. Radiography and clinical appearance of a 15-year old female AIS patient with a double curve of initially 32 degrees thoracic and 28 degrees lumbar. The lumbar curve has been fused although this leads to decompensation and clinical deterioration. The risk for curve progression in this relatively mature patient (15 years of age, three years postmenarchial) was minimal before undergoing surgery!

**Figure 2:**
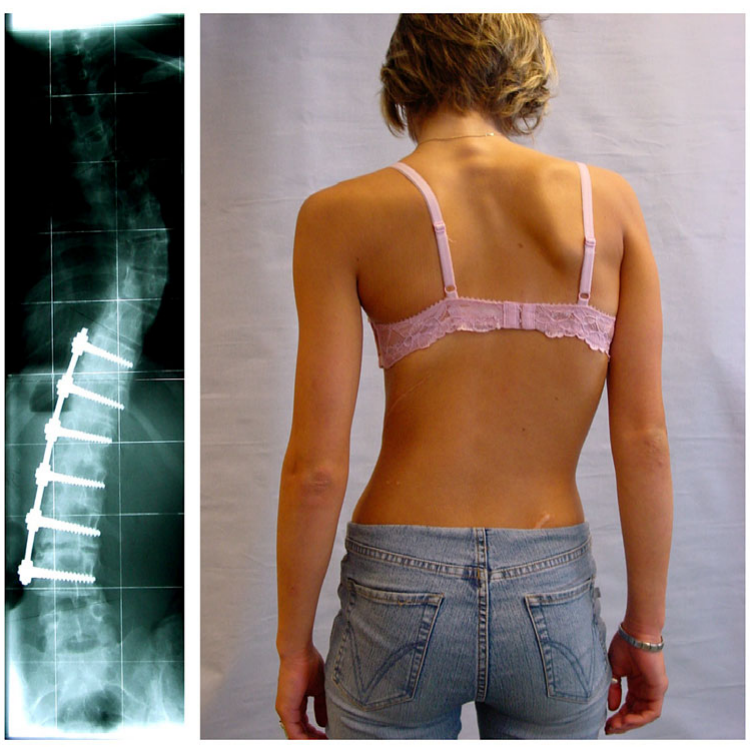
**Clinical deterioration after surgery for Adolescent Idiopathic Scoliosis (AIS) in another patient**. A different patient from the same centre as the  one shown on Fig. 1, with similar clinical result after surgery, emphasizing  that the case presented here is not unique.

Historically, the indication for surgery has been set to a curve measurement of 50° Cobb angle [4], but this limit of Cobb angle varies greatly within the surgical community and today in Germany patients with curves of less than 35° are operated upon (Fig. 1.). The patients can become concerned about developing health risks that do not always necessarily occur in patients with AIS. In actual fact, only a small percentage of the population of AIS patients operated on suffer in respect of their physical appearance enough to justify this very risky procedure, which may not even lead to any clinical improvement (Fig. 1 and 2). Today there is still no specific evidence that surgery can change the signs and symptoms of scoliosis [5].

AIS is a relative benign condition [6,7] and therefore the indication for surgery is clearly cosmetic or psychological [8]. In a recent publication [9], claims were made that there is a 'need for surgery', which in relation to the lack of evidence to support this seems somewhat questionable making it necessary to investigate indications for surgery in this specific population.

One might assume that the main benefit of surgery is a biomechanical correction. But when one realises that primary correction effects are not stable after surgery [10,11], not even within the first year post-surgery and that neither back shape nor self esteem may improve to a satisfactory level by the surgical intervention [12], a more scientific basis remains to be desired on the true outcome after surgery.

## Case presentation

A 15-year old female patient from Italy presented originally with a well-compensated double curve pattern; with a Cobb angle of 32° thoracic and 28° thoracolumbar (Fig. 1). This case certainly is not within the usual range that indicates surgical intervention,  with a curve which is not even  clinically evident.  

The patient was advised to undergo surgery to prevent possible future negative impacts of the long-term signs and symptoms of scoliosis upon her health. Considering this risk, the patient agreed to undergo the surgery, which was performed in one of Germanys leading centres for spinal surgery.

The patient was 15 years of age, 30 months postmenarchial. The thoracolumbar curve was corrected with fusion, using the ventral approach, while the thoracic curve, which clearly showed wedged vertebrae, known as structural scoliosis (Fig. 1), remained untreated.

This 15-year old patient was almost fully grown at the time the operation was performed. The postoperative x-rays measured as a 36/22° Cobb angle and presented one year after surgery with 52/28°. The minor curve was fused, the major curve has clearly progressed and the wedged disc space at L3/4 (Fig. 1) indicates that future problems may occur with degeneration and subsequent pain.

The operation produced an unbalanced appearance with radiological and clinical imbalance to the right. The clinical appearance of the patient has clearly deteriorated by the procedure performed; comparing the initial presentation of a well compensated double curve pattern and the post-surgical decompensated single curve pattern.

## Discussion

As pointed out by Goldberg and collaborators [8] spinal fusion in patients with AIS replaces one pathology (a curved spine) by another (stiff spine) and therefore the indication for corrective surgery in patients with AIS is mainly cosmetic. This paradigm is also supported by the fact that signs and symptoms of AIS cannot be changed by spinal fusion [5].

As this patient had no cosmetic complaints before undergoing surgery there was no indication in the case presented here.

Secondly it is questionable as to why in this case only the lumbar curve has been fused. The patient was told by the surgeon that lumbar scoliosis may lead to loss of lumbar lordosis, which might cause low back pain in adulthood. However, when there is no proof that spinal fusion may prevent pain [5], the procedure performed here is not justified.

In contrary, the operation lead to a clear imbalance of the trunk and one year after the operation and after a progression of 20° Cobb in the thoracic curve which had been left unfused, there is no doubt, that a secondary rebalancing of the thoracic curve never will occur.

With respect to surgery, no prospective controlled or randomised controlled studies support the use of this surgery in the treatment of AIS and as previously highlighted by Hawes [5], signs and symptoms of scoliosis cannot be changed by surgery.

One should respect the psychological indication for surgery, when a patient is unable to cope with the deformity. The assumption that there is an 'indication for surgery' [9], in spite of the known long-term risks [1,13,14], when utilised by someone in a highly responsible and ethical role should be used with caution, as it is merely an assumption and has no scientific body of research to support it.

Instead of achieving substantial evidence for surgical treatment on a higher level, as was the previous vision of Paul Harrington [15] and reporting the long-term complications of post-surgery to improve the safety for patients, the surgical community has presented a body of papers describing HRQL [5].

On the other hand there exists a large body of literature on the complications of surgery in patients with scoliosis. The most complete overview is given by Hawes [5], who in her review has found one study indicating that the long-term re-operation rate may be as high as 40%.

As pointed out by Hawes [5] risks of spinal fusion include those occurring in any major surgery such as severe blood loss; urinary infections due to catheterization; pancreatitis; and obstructive bowel dysfunction due to immobilization during and after surgery. Other scoliosis surgery risks are summarized below. The frequency of specific complications, including death, is unknown. In the U.S., for example, most states do not have mandatory reporting policies for 'serious physical injury' and even when reporting is mandatory, definitions, interpretation, and compliance vary. Therefore, as with other data on surgical outcome, information is based on voluntary reporting by clinicians who carry out the surgery.

In her review Hawes [5] lists the complications in the following order:

Death and neurological damage. Incidence of death as a complication of spine surgery, for otherwise healthy adolescent and adult patients, is reported to be <1%, but can as well be higher (20%) in the adult population undergoing surgery.

Symptoms of neurological damage include partial or total paraplegia, quadriplegia, or peripheral nerve deficit and can emerge a decade after surgery.

Loss of normal spinal function. In every patient who undergoes spinal fusion surgery, there is an irreversible loss of the normal ability of the spine to move. Spinal fusion is correlated with significantly reduced range of motion over the entire spine, including the nonfused segments [5,8].

Strain on unfused vertebrae. The fact that part of the spinal column is made rigid after spinal fusion means that added strain is placed on the unfused part of the skeletal framework as the patient attempts to carry out normal activities requiring spinal motion [5,8].

Post-surgery pain. Pain, which may develop soon after surgery or after 10 years or more, is the primary reason patients have to be operated on more than once [5].

Infection and inflammatory processes. Infections introduced into the spinal column during surgery may manifest months or years later [5].

Curvature progression. Some curvatures continue to progress after spinal fusion due to broken rods or other failure of instrumentation [5].

Decompensation and increased sagittal deformity. Reducing the lateral curvature in thoracic scoliosis can exacerbate the sagittal deformity and cause flattening of the cervical, thoracic and/or lumbar spine beyond that caused by the deformity itself [5].

Despite the application of force to straighten and derotate the spine during surgery, the rib hump can worsen after spinal fusion [10,11,16-20].

Other long-term complications. The complexity of spinal surgery is reflected in the diversity of complications that may result months or years later. Given the time delay and difficulty in diagnosis, it is likely that only a minority of such events are recognized as surgical complications. In one study 40% of the patients treated surgically as adults required salvage surgery within a follow-up period averaging 55 months after surgery [21].

Most of the papers reporting short-term complications of spinal fusion in patients with scoliosis are retrospective studies. Just recently a prospective study on complication has been published stating the rate of complications being much higher than expected from previous retrospective reviews [22]. Therefore also the true rate of complications may be higher in the long-term than the rate reported so far.

In view of the lack of evidence to demonstrate that signs and symptoms of scoliosis can be changed by the instrumentation and fusion of a curved spine [5], no specific indication for spinal surgery in the treatment of patients with AIS exists, other than the cosmetic or psychological indication. The decision to perform surgery should not be based upon a surgeons' opinion or assumption, but upon the well informed patients'. Conservative treatment options should be explained and offered to the patients [23-25] without prejudice.

The high rate of short and long-term risks [5], including the possibility of an unbalanced and cosmetic undesirably appearance after surgery as reported here (Fig. 1 and 2), should be clearly explained to the patients before surgery is decided upon as a treatment option.

## Conclusion

Scoliosis surgery is mainly indicated for cosmetic or psychological reasons. Therefore the procedure or treatment option that leads to the best possible clinical appearance and balance should to be chosen. Patients should be informed that surgery will not improve their health status. Clinical deterioration due to surgery is not yet introduced in the literature as a complication of scoliosis surgery. This complication however, should be presented to the patient when advising patients on treatment options, so that consent in the pre-surgical stages is actually sufficiently informed and to prevent such cases as the one presented within this paper.

In view of the high rate of complications, the limited gains to be  derived from spinal fusion should be assessed and clearly explained to  patients before the procedure is undertaken [14].

## Competing interests

The author declares to have no competing interests.
